# TLR7/8 agonist induces a post-entry SAMHD1-independent block to HIV-1 infection of monocytes

**DOI:** 10.1186/s12977-016-0316-3

**Published:** 2016-12-01

**Authors:** Henning Hofmann, Bénédicte Vanwalscappel, Nicolin Bloch, Nathaniel R. Landau

**Affiliations:** 1 Department of Microbiology, NYU School of Medicine, New York, NY USA; 2 Department of HIV and Other Retroviruses, Robert Koch Institute, Berlin, Germany

**Keywords:** TLR7/8, HIV restriction, R848, Monocytes, Restriction factor

## Abstract

**Background:**

Monocytes, the primary myeloid cell-type in peripheral blood, are resistant to HIV-1 infection as a result of the lentiviral restriction factor SAMHD1. Toll-like receptors recognize microbial pathogen components, inducing the expression of antiviral host proteins and proinflammatory cytokines. TLR agonists that mimic microbial ligands have been found to have activity against HIV-1 in macrophages. The induction of restriction factors in monocytes by TLR agonist activation has not been well studied. To analyze restriction factor induction by TLR activation in monocytes, we used the imidazoquinoline TLR7/8 agonist R848 and infected with HIV-1 reporter virus that contained packaged viral accessory protein Vpx, which allows the virus to escape SAMHD1-mediated restriction.

**Results:**

R848 prevented the replication of Vpx-containing HIV-1 and HIV-2 in peripheral blood mononuclear cells and monocytes. The block was post-entry but prior to reverse transcription of the viral genomic RNA. The restriction was associated with destabilization of the genomic RNA molecules of the in-coming virus particle. R848 treatment of activated T cells did not protect them from infection but treated monocytes produced high levels of proinflammatory cytokines, including type-I IFN that protected bystander activated T cells from infection.

**Conclusion:**

The activation of TLR7/8 induces two independent restrictions to HIV-1 replication in monocytes: a cell-intrinsic block that acts post-entry to prevent reverse transcription; and a cell-extrinsic block, in which monocytes produce high levels of proinflammatory cytokines (primarily type-I IFN) that protects bystander monocytes and T lymphocytes. The cell-intrinsic block may result from the induction of a novel restriction factor, which can be termed Lv5 and acts by destabilizing the in-coming viral genomic RNA, either by the induction of a host ribonuclease or by disrupting the viral capsid. TLR agonists are being developed for therapeutic use to diminish the size of the latent provirus reservoir in HIV-1 infected individuals. Such drugs may both induce latent provirus expression and restrict virus replication during treatment.

**Electronic supplementary material:**

The online version of this article (doi:10.1186/s12977-016-0316-3) contains supplementary material, which is available to authorized users.

## Background

Toll-like receptors (TLR) are membrane-associated pattern recognition receptors (PRRs) expressed in myeloid cells that serve as a line of first defense against bacterial and viral pathogens. The TLRs are activated by pathogen-associated molecular patterns (PAMPs) that trigger a signal transduction to activate innate and adaptive immune responses, resulting in the release of proinflammatory cytokines, chemokine and type-I interferons (IFN). The cytokines released induce the expression of an array of antiviral genes that inhibit virus replication through various mechanisms and cause T cells to increase expression of co-stimulatory proteins. PAMPs detected by the 10 TLRs encoded in the human genome include single-stranded RNA (TLR7 and 8), double-stranded RNA (TLR3) and double-stranded DNA (TLR9) [1–3].

Because of their ability to induce innate and adaptive responses, TLR agonists have been explored as antiviral therapeutic agents. Agonists for TLR3, TLR4, TLR7, TLR8 and TLR9 have been used in nonhuman primate models for dengue virus and in murine models for herpes simplex virus-type-1 and -2 where they have been found to attenuate viral symptoms and replication [4–6]. Several TLR agonists inhibit the replication of HIV-1 in lymphocytes, monocytes and monocyte-derived macrophages (MDM). The imidazoquinoline TLR7/8 agonists R848 (resiquimod) and gardiquimod were found to inhibit HIV-1 replication in MDMs by inducing soluble antiviral factors and in the case of gardiquimod, by acting as a nucleoside reverse transcriptase inhibitor [7, 8]. In monocytes isolated from HIV-infected individuals, R848-treatment reduced viral RNA in the culture supernatants [9]. In addition, the TLR9 agonist ODN2395 induces the restriction factors APOBEC3G and SAMHD1 [10]. Clinically, TLR7 and 8 agonists are used to treat inflammatory disorders and viral infections. R848 is used to treat psoriasis and HSV-2 induced genital lesions and the related imidazoquinoline, imiquimod, is used to treat human papilloma virus genital warts [11, 12].

 In myeloid cells in culture, HIV-1 has been reported to be sensed by PRRs including the TLRs [13]. In plasmacytoid dendritic cells (pDC), HIV-1 virion genomic RNA was found to activate endosomal TLR 7/8 and TLR9 resulting the production of high levels of type-I IFN  [14–16]. In addition, HIV-1 RNA activates TLR8, leading to inflammasome activation and the release of IL-1β and IL-18 [17, 18]. Oligonucleotides with HIV-1 RNA derived sequences stimulate TLR7 and TLR8 in pDCs, monocyte-derived DCs (MDDC), MDMs and monocytes [19, 20]. In addition, HIV-1 virions and TLR7/8 agonists both stimulate monocytes to produce IP-10 [21]. The HIV-1 envelope glycoprotein activates cell surface TLR2 and TLR4 [22, 23]. While these data are based upon studies of cells in culture, the role of innate sensing in HIV-1 replication and pathogenesis in vivo remains to be determined.

Monocytes, the myeloid cell-type that is most prevalent in the blood, are largely resistant to HIV-1 [24] as a result of SAMHD1, a phosphohydrolase that depletes the intracellular pool of dNTPs, preventing reverse transcription [25–27]. HIV-2 and the SIV of rhesus macaques encode Vpx, a virion-packaged accessory protein that induces the proteasomal degradation of SAMHD1 in the nucleus, allowing the viruses to evade the restriction and replicate in monocytes, macrophages and DC [28–34]. HIV-1 lacks Vpx accounting for the inability of the virus to replicate to high titer on monocytes, macrophages and DC. Vpx is packaged in HIV-2 and SIV virions by an amino acid motif located at amino acids 17–26 in the P6 region of the Gag polyprotein precursor that is absent in HIV-1 [35]. The virus can be engineered to package Vpx by placing the SIV Vpx packaging motif in Gag P6, resulting in a two order of magnitude increase in infectivity of myeloid cells [36].

In this study, we investigated the effect of the TLR7/8 agonist R848 on HIV-1 replication in primary myeloid and lymphoid cells using Vpx-containing HIV-1 and HIV-2. The agonist potently restricted the replication of both viruses in monocytes. The restriction was not mediated by SAMHD1 or other known restriction factors including APOBEC3A. The restriction was post-entry, preventing reverse transcription and was independent of type-I IFN signaling. The restriction was associated with destabilization of the virion genomic RNA of the in-coming virus suggesting a role for a restriction factor with ribonuclease activity or with an ability to interfere with capsid stability. In addition to inducing a novel IFN-independent restriction factor, R848 caused monocytes to release proinflammatory cytokines that protected bystander monocytes and activated CD4+ T cells from HIV-1 infection.

## Methods

### Cell culture

293T and HL116 cells were cultured in Dulbecco’s modified Eagle medium (DMEM) supplemented with 10% fetal bovine serum (FBS) and penicillin/streptomycin. PBMC were prepared by Ficoll density gradient centrifugation of blood from anonymous donors provided by the New York Blood Center. The monocytes were purified by plastic adherence and cultured in RPMI containing 10 mM HEPES, 24 µg/ml gentamicin, and 5% heat inactivated pooled human serum (PHS). Monocyte purity was determined by flow cytometry using a PE-conjugated anti-CD14 antibody (BD biosciences). MDDC were prepared by culturing the monocytes for 5 days in medium containing 110 U/mL GM-CSF and 300 U/mL IL-4, replacing the cytokines every 2 days. MDM were prepared by culturing the monocytes for 5 days in medium containing 50 ng/ml GM-CSF. CD4+ T cells were isolated by negative selection of the PBMCs on EasySep human CD4+ T cell magnetic beads (Stem Cell Technologies). The CD4+ T cells were activated for 2 days with CD3/CD28 beads (LifeTechnologies) using 2 cells per bead and cultured in RPMI containing 5% PHS, 1 mM HEPES, 2 mM l-glutamine, MEM non-essential amino acids, penicillin/streptomycin and 100 U/mL recombinant IL-2. CD4+ T cell purity was evaluated by staining with Alexa 700-anti-CD3 and allophycocyanin-anti-CD4 monoclonal antibodies (BioLegend) and analyzed by flow cytometry. BMDCs were prepared by flushing the marrow cells from C57BL/6 SAMHD1 knock-out mouse femurs. The red blood cells were removed with ammonium chloride lysing buffer and the cells were cultured for 6–8 days in RPMI supplemented with 1 mM sodium pyruvate, 2 mM l-glutamine and 10 ng/ml murine GM-CSF (Peprotech). R848 to stimulate TLR7/8 was obtained from Invivogen and diluted to the indicated final concentration(s) in the respective culture medium.

### Plasmids

HIV-2 GFP reporter virus plasmids pHIV2.GFPΔVpr and pHIV2.GFPΔVprΔVpx were constructed by subcloning the BsmB-I to Avr-II fragment of pHIV2.E-.GFP that contains the *vpx*/*vpr* genes. Translational stop codons were introduced into *vpx* at amino acids 22 and 24 without altering the overlapping amino acids encoded by *vif*. The *vpr* translational initiation codon was removed by mutation to ATC and amino acids 2, 3 and 4 were changed to translational termination codons by overlapping PCR (TAA TAA TGA). The amplicon was digested with BsmBI and Avr-II and cloned back into pHIV2.E-.GFP. Plasmid sequences were confirmed by nucleotide sequence analysis.

### Viruses

Reporter viruses were prepared by calcium phosphate transfection of 293T cells. VSV-G pseudotyped HIV-1 luciferase reporter virus was produced by co-transfecting with pNL.luc3.p6* E-R- [36], pVSV-G [37] and pcVpx [36] or pcDNA6 at a mass ratio of 10:1:1. Viruses bearing CCR5-tropic HIV-1 envelope glycoprotein from transmitted founder virus WEAUd15.410.5017 [38] were produced by transfection with a mass ratio of 6:6:1. HIV-1 GFP reporter virus was produced by cotransfection with pHIV1.CMV.GFP.p6* E-R- [39]. HIV-2 GFP reporter virus was produced by cotransfection with pHIV2.GFP E-R- or pHIV2.GFP E-R-X- and pVSV-G at a mass ratio of 10:1. Viruses were harvested 48 h post transfection, filtered through a 0.45 µm filter and concentrated by ultracentrifugation for 90 min at 4 °C at 30,000 rpm through a 20% sucrose cushion. The viruses were resuspended in RPMI containing 5% PHS, frozen at −80 °C and tittered on 293T cells.

### qPCR quantification of HIV-1 reverse transcripts

Monocytes were isolated by plastic adherence from 1.5 × 10^7^ PBMC plated in 6-well plates. The cells were treated with 10 μM R848 or 100 U/mL IFNα. After 24 h, the cells were infected with benzonase-treated Vpx-containing HIV-1 luciferase reporter virus (1.5 × 10^7^ cps) in the presence or absence of 10 μM nevirapine. At 40 h post-infection, DNA was isolated and 250 ng were analyzed by qRT-PCR using SYBR green (Molecular Probes) with primers that amplified early or late HIV-1 reverse transcripts (early RT: fw 5′-GTG CCC GTC TGT TGT GTG AC and rev 5′-GGC GCC ACT GCT AGA GAT TT; late RT: fw 5′-TGT GTG CCC GTC TGT TGT GT and rev 5′-GAG TCC TGC GTC GAG AGA GC) [40]. The data were normalized to a standard curve generated with proviral plasmid DNA serially diluted in 293T cell genomic DNA.

### Reverse transcriptase qRT-PCR mRNA quantification

RNA was isolated from 5.0 × 10^7^ monocytes using Trizol and treated with RNase-free DNase I (Roche). cDNA was synthesized using an oligo-dT primer and Transcriptor RT (Roche). cDNA corresponding to 50 ng of RNA was analyzed by qRT-PCR using SYBR green to quantify mRNA transcripts for p21 (fw 5′-GCA GAC CAG CAT GAC AGA TTT and rev 5′-GGA TTA GGG CTT CCT CTT GGA), RRM2 (fw 5′-AAG AAG AAG GCA GAC TGG GC and rev 5′-CCA GGC ATC AGT CCT CGT TT) IL-1β (fw 5′-ATG ATG GCT TAT TAC AGT GGC AA and rev 5′-GTC GGA GAT TCG TAG CTG GA-) and GAPDH (fw 5′-TGG AAG GAC TCA TGA CCA CAG and rev 5′-CAG TCT TCT GGG TGG CAG TGA). Reactions without RT were included to control for genomic DNA contamination. The ΔΔCT relative to GAPDH in the untreated samples was set to 1.

### βlam fusion assay

Virions containing Vpr-β-lactamase fusion protein (Vpr.BlaM) were produced using Vpr.BlaM expression vector pMM310. Monocytes (1.0 × 10^6^) were treated for 24 h with R848 and then exposed to the virions. After 5 h, the cells were loaded with coumarin cephalosporin fluorescein (CCF2) (ThermoFisher) and analyzed by flow cytometry.

### SAMHD1 degradation

Monocytes (5.0 × 10^6^) were incubated with 10 μM R848 or 100 U/mL IFNα for 24 h and then infected with Vpx-containing HIV-1 (5.0 × 10^6^ cps). The next day, the cells were lysed in buffer containing 50 mM HEPES, 150 mM KCl, 2.0 mM EDTA, 0.5% NP-40 and protease inhibitors. The cell lysates were analyzed on an immunoblot probed with anti-SAMHD1 mAb (Origene) and anti-GAPDH mAb (Ambion) and horseradish peroxidase-goat anti-mouse immunoglobulin (Pierce) second antibody. The signals were detected with Super Signal West Pico (ThermoFisher) and quantified on an Odyssey FC imager (LI-COR).

### Cell viability assays

Cell viability was determined by trypan blue staining, MTS assay (Promega) and flow cytometry with Pacific Blue fixable viability dye eflour450 (eBiosciences) staining.

### Cytokine quantification

Monocytes were treated for 2 h with 1 μM R848 after which the drug was removed by extensive washing. After 22 h, the supernatant was harvested and type-I IFN was quantified on HL116 IFN reporter cells [41] and the proinflammatory cytokines were quantified by cytometric bead array (BD Biosciences).

### RNA stability assay

Monocytes (2 × 10^7^) in a 6-well plate were untreated or treated for 24 h with 1 μM R848. 10 μM Nevirapine was added and the cells were infected for 2 h at 16 C° by spinnoculation at 2200 rpm in a table-top centrifuge with Vpx-containing HIV-1 (2 × 10^7^ cpm luciferase activity). The cells were harvested 0.5, 1 and 3 h post-infection and RNA was prepared. The HIV-1 genomic RNA copies in 175 ng RNA was quantified by SYBR green reverse transcriptase qRT-PCR using primer pairs that amplified the reporter virus luciferase gene (luc3-fw3 5′-TGG GCG CGT TAT TTA TCG GA and luc3-rv3 5′-GCT GCG AAA TGC CCA TAC TG), the 5′ end of the viral RNA (5′-LTR-fw 5′-GCT CAA AGT AGT GTG TGC CC, 5′-LTR-rev 5′-CTC CTC TGG CTT TAC TTT CGC, and the 3-end of the viral RNA (3′-LTR-fw 5′-CGA GCT TGC TAC AAG GGA C and 3′-LTR-rv 5′-GCT TAA GCA GTG GGT TCC C). Control reactions without RT were included to detect contaminating viral DNA. HIV genomic RNA was calculated as ΔΔCT relative to GAPDH mRNA.

## Results

### R848 induces a block to HIV-1 infection of monocytes

**Fig. 1:**
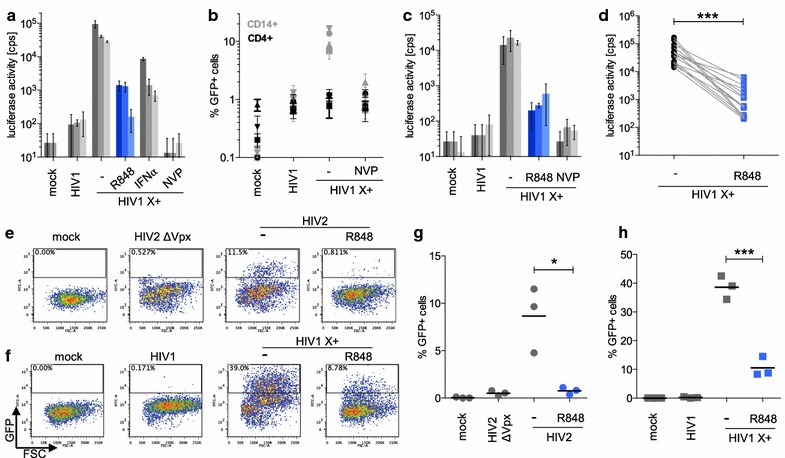
R848 prevents the infection of monocytes by HIV-1 and HIV-2. **a** Non-stimulated PBMCs from three donors (*one bar* represents one donor) were untreated (−) or treated for 24 h with 10 µM R848 (*blue*) or 100 U/mL IFNα and then infected with HIV-1 luciferase reporter virus lacking (HIV1) or containing packaged Vpx (HIV1 X+). Uninfected (mock) or nevirapine (NVP) treated cells were included as controls. After 72 h luciferase activity was measured. **b** Non-stimulated PBMCs were infected with HIV-1 GFP reporter virus containing (HIV1 X+) or lacking (HIV1) Vpx. After 72 h the cells were stained with Alexa700-anti-CD4, APC-anti-CD3 and PE-anti-CD14 antibodies and analyzed by flow cytometry. **c** Monocytes from three donors were treated with R848 (*blue*) and infected with reporter virus as in (**a**). **d** Monocytes from 15 healthy donors were treated (*blue squares*) or untreated (*black dots*) with 10 µM R848 and then infected with Vpx-containing luciferase reporter virus (***p = 0.0004). **e**, **f** Monocytes were treated or untreated with R848 (−) for 24 h and then infected with wild-type or delta Vpx HIV-2 GFP reporter virus (HIV2) (**e**) or Vpx-containing (HIV1 X+) or lacking (HIV1) HIV-1 GFP reporter virus (**f**). Uninfected cells serve as control (mock). After 72 h, the cells were analyzed by flow cytometry. **g** Summary of 3 healthy donors infected as in **e**; *p = 0.0175. **h** Summary of 3 healthy donors infected as in (**f**); ***p = 0.0008

The TLR7/8 agonist R848 has been found to induce a block to HIV-1 replication in monocytes and MDMs [7, 8, 10]. To further investigate the mechanism by which the drug interferes with HIV-1 replication we used a VSV-G-pseudotyped reporter virus engineered to package the SIVmac accessory protein Vpx [36]. Vpx degrades SAMHD1, overcoming the block to HIV-1 infection of resting T cells and primary monocytes [33, 34, 42, 43] allowing us to test how well the drug blocks infection and to investigate its antiviral mechanism. To determine which cell-types are protected by the agonist, we treated PBMCs from healthy donors for 24 h with R848 and then infected them with Vpx-containing, HIV-1 luciferase reporter virus. For comparison, we treated the PBMCs with type-I IFN, an inducer of multiple antiviral pathways. To ensure that the activity measured was due to *bona fide* infection, control PBMCs were treated with the reverse transcriptase inhibitor nevirapine. Infectivity was measured by intracellular luciferase assay 72 h post-infection. The results showed that R848 potently blocked infection of the PBMCs, with 10 μM drug reducing infectivity 20- to 175-fold in 18 different donors (Fig. 1a and Additional file 1). This effect was at least as strong as a high dose of IFNα. The infectability of PBMCs was highly dependent upon Vpx in the virions which increased the infection by 200-fold.

PBMCs are a mixture of resting lymphocytes and myeloid cell-types. Because Vpx facilitates the infection of monocytes and resting lymphocytes, it was unclear which cell-types in the PBMC population had become infected. To determine the cell-types that had been infected by Vpx-containing virus, we infected PBMCs with VSV-G pseudotyped, Vpx-containing HIV-1 GFP reporter virus and after 3 days, analyzed the cells by flow cytometry to determine the numbers of infected CD4+ T cells (CD4+/CD3+/CD14−) and monocytes (CD4−/CD3−/CD14+). The results showed that the infection was Vpx-dependent and that it was the monocytes (CD14+) that had been infected (Fig. 1b). Despite the packaged Vpx, the resting CD4+ T cells were not appreciably infected. We concluded that Vpx allowed for efficient infection of the monocytes and that R848 blocked their infection.

To confirm that it was the monocytes that had been protected by R848 and to test whether this was a direct effect or whether it was mediated by another cell-type in the culture such as pDCs that had released a soluble inhibitor, we tested the effect of R848 on monocytes purified from the PBMCs of three healthy donors. The results showed that the drug protected the monocytes from infection (Fig. 1c). Further analysis of monocytes purified from 15 donor PBMCs showed that the drug was active on monocytes from all of the donors, reducing the number of infected cells 15- to 100-fold (Fig. 1d). The effect of the drug was not the result of toxicity as an analysis using three cell viability assays showed no effect on cells treated with R848 at concentrations that induced the block to infection (Additional file 2).

The experiments above used virions engineered to package Vpx. To determine whether R848 would block the infection of monocytes by a virus for which these cells are a natural target and that naturally encodes Vpx, we tested its effect on infection by HIV-2. We infected monocytes with wild-type or Vpx-mutated HIV-2 GFP reporter virus. For comparison, the monocytes were infected with Vpx-containing or lacking HIV-1 GFP reporter virus and after 3 days analyzed infectivity by flow cytometry. The results showed that infection of the monocytes by HIV-2 was dependent upon Vpx and that the infection was blocked by R848 (Fig. 1e, g). The effect on HIV-2 was somewhat more pronounced than on HIV-1 (Fig. 1f, h), perhaps because of the decreased infectivity of this virus. For both viruses, the drug decreased the number of GFP+ cells and not the mean fluorescence intensity, indicating that the drug prevented infection rather than decreased LTR-driven transcription of the GFP reporter. The findings demonstrate that R848 induces a block to HIV-2 in monocytes, a cell-type that is a natural target of the virus.

### The R848-induced restriction is SAMHD1-independent

**Fig. 2:**
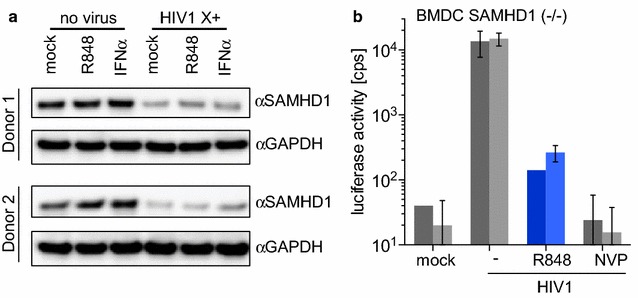
R848 induces a SAMHD1-independent block to infection of monocytes. **a** Monocytes from 2 healthy donors were treated for 24 h with 10 µM R848 or 100 U/mL IFNα and then infected with Vpx-containing HIV-1 (HIV1 X+). After 24 h, lysates were prepared and SAMHD1 was quantified by immunoblot analysis with a GAPDH loading control. **b** BMDC from two SAMHD1 knock-out mice (*one bar* per mouse) were treated for 24 h with (*blue*) or without 10 µM R848 and then infected with HIV-1 luciferase reporter virus (HIV1) in the presence or absence of Nevirapine (NVP). Luciferase activity was measured after 72 h

SAMHD1 is expressed in monocytes where it acts as the major block to infection, as is clear from the ability of Vpx-containing viruses to efficiently infect these cells. The fact that R848 blocked the infection of monocytes with Vpx-containing virus suggests that SAMHD1 was not the mechanism by which R848 protected these cells from infection. However, this finding does not definitively rule-out a role for SAMHD1-mediated restriction, as R848 could prevent SAMHD1 degradation by Vpx or activate its phosphohydrolase activity. To test whether the R848-induced block to infection was mediated by SAMHD1, we treated monocytes with the drug for 24 h and then infected them with Vpx-containing HIV-1 or control supernatant. The next day we quantified SAMHD1 protein by immunoblot analysis. R848 did not affect the amount of SAMHD1 nor did it interfere with the ability of Vpx to induce SAMHD1 degradation (Fig. 2a). To determine whether R848 inhibition was dependent on SAMHD1, we tested whether the drug blocked the infection of SAMHD1 knock-out mouse bone marrow derived DCs (BMDC). R848 treatment caused a 100-fold block to infection of the knock-out BMDCs by HIV-1 reporter virus, an effect that was similar to that in human monocytes (Fig. 2b). The drug had no effect on the viability of the BMDCs (Additional file 2).

### R848 acts rapidly and is active against HIV-1 bearing a primary CCR5-using HIV-1 envelope glycoprotein

**Fig. 3:**
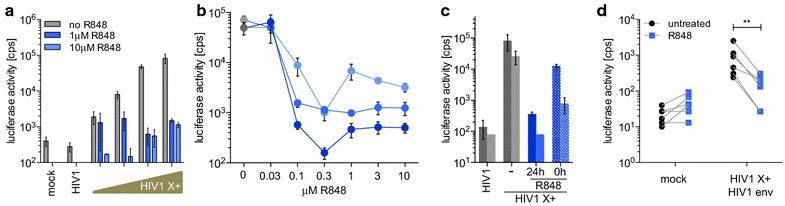
R848 induces a potent and rapid block to infection with virus bearing a primary HIV-1 isolate envelope glycoprotein. **a** Monocytes were incubated with 1 µM (*dark blue*) or 10 µM (*light blue*) R848 for 24 h and then infected with increasing amounts of Vpx-containing HIV-1 luciferase reporter virus (0.1, 0.3, 1.0 and 3 × 10^6^ cps). Uninfected (mock) and cells infected with virus lacking Vpx (HIV1) were included as controls. **b** Monocytes from three donors were treated for 24 h with serial dilutions of R848 and then infected with Vpx-containing HIV-1 luciferase reporter virus. **c** Monocytes were untreated (−) or treated with 10 µM R848 24 h before infection (24 h, *blue solid*) or at the time of infection (0 h, *blue dotted*) with Vpx-containing or lacking HIV-1 luciferase reporter virus. **d** Monocytes from 6 healthy donors were treated for 24 h with R848 (*blue squares*) or untreated (*black dots*) and then infected with Vpx-containing HIV-1 luciferase reporter virus bearing a CCR5-tropic transmitted founder envelope glycoprotein (HIV1+ HIV1 env) or uninfected (mock). **p ≤ 0.01 In all 4 experiments **a**–**d** luciferase activity was measured 72 h post-infection

To characterize the antiviral activity of R848, we tested whether the effect could be saturated by increasing the MOI, as is the case for some of the other restriction factors by treating monocytes with 1 or 10 μM R848 and infecting with increasing MOI. The results showed that the degree of inhibition remained constant regardless of the MOI (Fig. 3a). Similar results were obtained with an analysis of two additional donors (Additional file 3). A dose–response curve for the drug on monocytes from three donors showed an average IC_50_ of 0.05 μM (Fig. 3b). The drug was most active when added 24 h prior to infection, causing a 100-fold reduction of HIV-1 reporter virus infection. When added at the same time as the virus, the block was still active, causing a 6.5- and 33-fold inhibition of infection in the two donors tested (Fig. 3c). To show that the restriction is not limited to VSV-G pseudotyped virus, we tested whether R848 would inhibit infection by reporter virus pseudotyped with a transmitted-founder envelope glycoprotein [38]. The results showed that virus pseudotyped by an HIV-1 envelope glycoprotein was senstive to the drug, resulting in a 2.5- to 8.5-fold reduction in the number of infected monocytes (Fig. 3d). While the reduction in infectivity was not as pronounced as that of the VSV-G pseudotype, this most likely results from the reduced infectivity of the reporter virus bearing HIV-1 envelope glycoprotein. The effect of which is a reduced dynamic range of the analysis, and does not reflect an ability of the virus to evade the R848-induced restriction. Taken together, R848 is a potent inhibitor of monocyte infection by HIV-1 that acts regardless of the route of entry and is not saturated by a high dose of virus.

### The block to infection is post-entry at reverse transcription

**Fig. 4:**
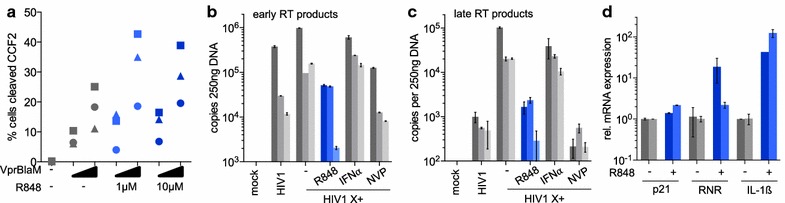
The block is post-entry at reverse transcription and independent of p21 and RNR. **a** Monocytes from 3 healthy donors (*dot*, *square*, *triangle*) were treated with the indicated R848 concentrations (*blue*) and infected (VprBlam) or uninfected (−) with two different amounts of Vpr.Blam-containing HIV-1. The ability of the cells to support virus fusion was measured by flow cytometry. **b**, **c** Monocytes from 3 healthy donors (*one bar per donor*) were treated with 10 µM R848 (*blue*), 100 U/mL IFNα or untreated (–) and 24 h later infected with Vpx-containing HIV-1 reporter virus (HIV1 X+). Uninfected (mock) or Nevirapine treated cells and cells infected with HIV-1 reporter virus lacking Vpx (HIV1) served as controls. 40 h post-infection, DNA of infected cells was isolated. Early (**b**) and late (**c**) HIV-1 reverse transcripts were quantified by RT-qPCR. **d** Monocytes from two donors (*one bar per donor*) were treated (+, *blue*) or untreated (−, *grey*) for 24 h with 10 μM R848. RNA was isolated and p21, RRM2 and IL-1β mRNA transcripts were quantified by qRT-PCR and normalized to GAPDH

To determine the step at which R848 blocks HIV-1 infection, we tested whether the drug prevents envelope glycoprotein-mediated fusion using the Vpr.βlaM fusion assay [44]. The results showed that R848 did not affect VSV-G-mediated fusion on monocytes (Fig. 4a). To test the effect of the drug on reverse transcription, we treated monocytes with R848, IFNα or nevirapine and then infected them with Vpx-containing HIV-1. After 40 h, we quantified the early and late HIV-1 reverse transcripts by qRT-PCR. The results showed that R848 decreased the copy number of early reverse transcripts 2- to 100-fold (Fig. 4b) and the number of late reverse transcripts 10- to 200-fold (Fig. 4c). IFNα had no effect on the number of viral DNA copies, consistent with its post-reverse transcription effect. The nevirapine control confirmed that the DNA copies were the result of reverse transcription. We concluded that R848 induces a block to reverse transcription and that the effect differs from that of type-I IFN.

We also considered the possibility that the antiviral activity was mediated by APOBEC3A, an antiviral host factor that has been proposed to restrict HIV-1 replication in monocytes [45, 46]. Immunoblot analysis showed that R848 did not induce the expression of APOBEC3A in monocytes (Additional file 4). We further considered the possibility that R848 acts on p21, which is thought to regulate the intracellular dNTP concentration by decreasing the expression of ribonucleotide reductase [47] or by regulating SAMHD1 activity [48]. However, qRT-PCR analysis showed R848 did not affect the abundance of p21 mRNA transcripts in monocytes and that it increased rather than decreased the number of RNR mRNA transcripts (Fig. 4d). Taken together, the findings demonstrate that the SAMHD1-independent block is post-entry at reverse transcription and is not mediated by APOBEC3A or p21 expression.

### R848 destabilizes HIV-1 virion genomic RNA

**Fig. 5:**
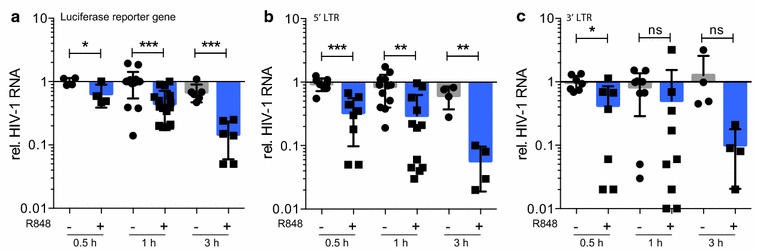
The R848-induced block to infection is associated with post-entry destabilization of HIV-1 genomic RNA. Monocytes from healthy donors were untreated (−, *grey*) or treated with 1 μM R848 (+, *blue*) for 24 h after which the cells were infected with HIV-1 luciferase reporter virus in the presence of nevirapine (NVP). The cells were harvested 0.5, 1 and 3 h post-infection and total RNA was prepared. The HIV genomic RNA copy number was quantified by reverse transcriptase RT-qPCR using primers specific for the luciferase reporter gene (**a**), the 5′ LTR (**b**) or the 3′ LTR (**c**). The viral RNA copies were normalized to GAPDH mRNA and the copy number in untreated cells harvested 0.5 h post-infection was set to 1. *p ≤ 0.05, **p = <0.001, ***p = 0.0005 and *ns* not significant

The effect on viral DNA copy number could result from a block to reverse transcriptase or from degradation of the viral genomic RNA template. To test for an effect of R848 on viral genomic RNA stability, we treated monocytes with the drug and then infected monocytes with HIV-1 luciferase reporter virus. To synchronize the infection, the cells were spinnoculated and to prevent degradation of the viral genomic RNA by reverse transcriptase RNaseH, nevirapine was added. At time points shortly after infection, the cultures were harvested and the viral RNA copy number was quantified by RT qRT-PCR. The results showed that over the course of the experiment R848 caused a decrease in the number of viral genomic RNA copies (Fig. 5a). RT qRT PCR analysis using primers specific for the 5′ or 3′ ends of the viral RNA showed that both ends of the RNA were lost with similar kinetics (Fig. 5b, c). The results suggest that the block to reverse transcription is associated with degradation of the viral RNA and that the degradation occurs at both ends of the molecules.

### R848 is specific for monocytes and MDM

**Fig. 6:**
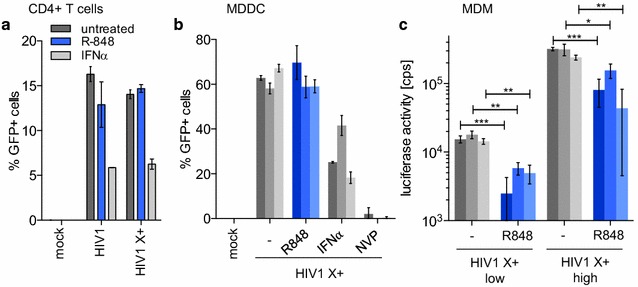
R848 does not prevent the infection of CD4+ T cells and MDDC. **a** Activated CD4+ T cells from 2 healthy donors were treated with 10 µM R848 (*blue*) or 100 U/mL IFNα (*light grey*) or untreated (*dark grey*) for 24 h and then infected with Vpx-containing (HIV1 X+) or lacking (HIV1) HIV-1 GFP reporter virus or with control supernatant (mock). Infectivity was quantified as percent GFP+ cells 72 h post-infection. **b** MDDC from 3 healthy donors (*one bar per donor*) were incubated with 10 µM R848 (*blue*) or 100 U/mL IFNα for 24 h or untreated (−). The cells were then infected with Vpx-containing (HIV1 X+) HIV1 GFP reporter virus. Uninfected cells (mock) and Nevirapine (NVP) treated cells served as controls. **c** MDM from 3 healthy donors (*one bar per donor*) were pretreated for 24 h with 10 µM R848 (*blue*) or untreated and then infected with 3 × 10^4^ (low) or 3 × 10^5^ (high) cps of Vpx-containing HIV1 luciferase reporter virus. Luciferase activity was measured 72 h post-infection. *p ≤ 0.05, **p ≤ 0.01 and ***p ≤ 0.001

To determine the cell-type specificity of R848, we tested it on activated CD4+ T cells, MDDC and MDM and compared its effect to that of IFNα. R848 did not significantly protect activated CD4+ T cells or MDDCs from infection (Fig. 6a, b) but decreased the infection of MDMs 3 to sixfold (Fig. 6c). The effect on MDM was significant but less pronounced than the effect on monocytes (compare to Fig. 1). These results demonstrate that R848-induced blocked to HIV-1 infection is most pronounced in monocytes.

### The block to a single-round infection is independent of type-I IFN or IL-1β

**Fig. 7:**
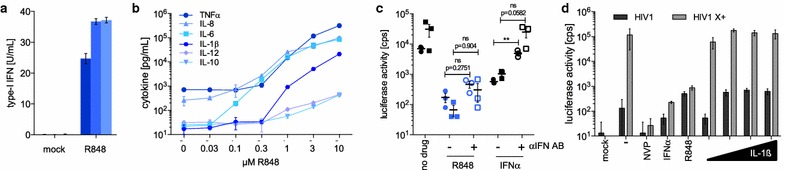
The block to single-cycle infection induced by R848 is not caused by IFN. **a** Monocytes from 3 healthy donors (*one bar per donor*) were untreated (mock) or treated with 10 µM R848-treated (*blue*) and after 24 h, the culture medium was collected and type-I IFN was quantified by bioassay. **b** Monocytes were incubated with different indicated concentrations of R848. After 24 h, the supernatant was collected and proinflammatory cytokines were quantified by cytokine bead array. **c** Monocytes from 2 healthy donors (*dot* and *square*) treated with 10 µM R848 (*blue*) or 100 U/mL IFNα with (*open symbol*) or without (*closed symbol*) IFN blocking antibody cocktail (±αIFN AB) were infected with Vpx-containing HIV-1 luciferase reporter virus. Luciferase activity was measured 72 h post-infection. **p ≤ 0.01 (**d**) PBMC were treated for 24 h with 100 U/mL IFNα, 10 μM R848 or 0.1, 1, 10 or 100 ng/mL IL-1β after which the cells were infected with Vpx-containing (HIV1 X+) or lacking (HIV1) HIV-1 luciferase reporter virus. Luciferase activity was measured 72 h post-infection

R848 could act directly on the target cells or could induce the production of soluble factors that provoke cellular innate immune defenses by both autocrine and paracrine mechanisms. To determine which proinflammatory cytokines are induced by R848, we treated monocytes for 24 h with increasing concentrations of the drug and then measured type-I IFN, IL-1β, IL-6, IL-8, IL-10, IL-12 and TNFα in the culture medium. The results showed that R848 strongly induced type-I IFN as measured in a biological assay (Fig. 7a). In addition, R848 induced the release of the other cytokines tested starting with as little at 0.3–1 μM R848, a concentration similar to that which induced the block to monocyte infection (Fig. 7b). Similar results were obtained for 2 additional donors (Additional file 5). TNFα, IL-1β, IL-6 and IL-8 were induced by more than a 100-fold.

Among the cytokines induced, type-I IFN and IL-1β are known inhibitors of HIV-1 replication. To determine whether type-I IFN played a role in the inhibition, we incubated monocytes with a cocktail of antibodies to IFNα, IFNβ and the type-I IFN receptor and then treated the cells with R848. The following day, we infected the cells with Vpx-containing HIV-1 luciferase reporter virus. To test the effectiveness of the antibody cocktail, IFNα was added to control cultures without R848. The results showed that R848 maintained its ability to inhibit infection in the presence of the antibody cocktail (Fig. 7c). The ability of the antibody cocktail to neutralize the antiviral effect of IFNα was confirmed in the control cultures. To test for a role for IL-1β in the R848-induced protection, we incubated PBMC with increasing concentrations of IL-1β for 24 h and then infected the cells with Vpx-containing or lacking HIV-1 luciferase reporter virus. IL-1β did not block the infection of PBMC (Fig. 7d and Additional file 6). Thus, the antiviral activity of R848 in a single-cycle of infection was not mediated by IFNα or IL-1β. This result does not rule-out the possibility that the cytokines released affect subsequent rounds of virus replication.

### R848 treatment of monocytes protects bystander cells

**Fig. 8:**
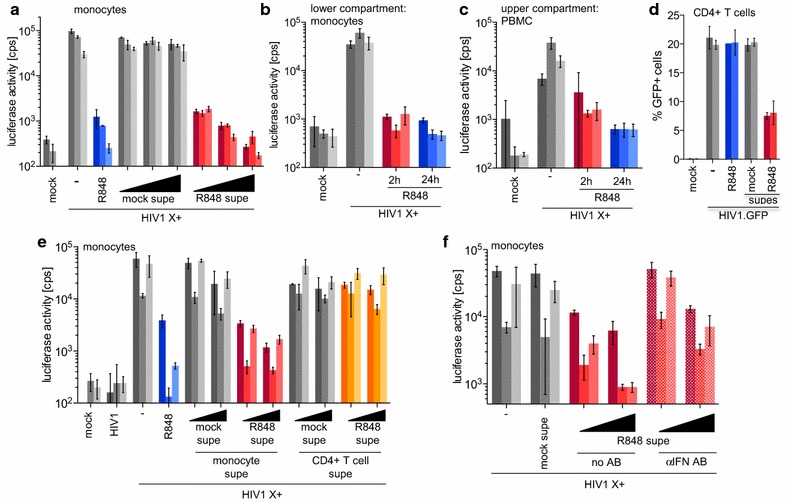
IFN released by R848-treated monocytes protects bystander cells from HIV-1 infection. **a** Monocytes from three healthy donors (*one bar per donor*) were incubated for 24 h with supernatant harvested from untreated (mock supe, *grey*) or R848-treated monocytes (R848 supe, *red*). Untreated (−) and cells pretreated with 1 µM R848 (R848, *blue*) served as controls. The cells were then infected with Vpx-containing HIV1 luciferase reporter virus (HIV1 X+) or uninfected (mock). Luciferase activity was measured 72 h post-infection. **b**, **c** Monocytes from 3 healthy donors treated with R848. The drug was either removed after 2 h (2 h, *red*) or left on the cells for 24 h (24 h, *blue*). The treated monocytes were placed in the lower compartment of a transwell plate (**b**) and PBMC were placed in the upper compartment (**c**). 24 h after R848 treatment, the cells in both compartments were infected with Vpx-containing HIV-1 luciferase reporter virus (HIV1 X+). (**d**) Activated CD4+ T cells from 2 donors were treated for 24 h with supernatant from untreated (mock supe), R848-treated monocytes (R848 supe, *red*) or with 1 μM R848 (R848, *blue*) and then infected with HIV-1 GFP reporter virus, or uninfected (mock). After 72 h, the number of GFP+ cells was determined by flow cytometry. **e** Monocytes from 3 healthy donors were treated for 24 h with supernatant from untreated (mock supe, *grey*) or R848-treated (R848 supe) monocytes (monocyte supe, *red*) or activated CD4+ T cells (CD4+ T cell supe, *orange*) and then infected with Vpx-containing HIV1 luciferase reporter virus. Controls without supernatant pretreatment (−) or treated with R848 (*blue*) were included. **f** Monocytes from 3 healthy donors were incubated with increasing amounts of supernatant from R848-treated monocytes (R848 supe, *red*) in the absence (no AB, *solid bars*) or presence of IFN blocking antibody cocktail (αIFN AB, *dotted bars*). The cells were infected 24 h post-treatment with Vpx-containing HIV-1 luciferase reporter virus

While IL-1β and IFNα are not involved in the block to single-cycle infection, it is possible that cytokines released in response to R848 protect bystander monocytes as well as T cells which themselves do not respond to R848. To test for bystander protection, we prepared conditioned medium from R848-treated monocytes, transferred it to fresh monocyte cultures and then infected the cells with Vpx-containing HIV-1 luciferase reporter virus. The results showed that the conditioned medium from R848-treated monocytes protected the monocytes from becoming infected (Fig. 8a). Similar results were obtained in a transwell experiment in which monocytes were seeded in the bottom compartment and treated with R848 for 2 h. The drug was washed out, unstimulated, autologous PBMC were seeded in the top compartment and cells in both compartments were infected with Vpx-containing HIV-1 luciferase virus. The results showed that the monocytes in the bottom chamber and the PBMC in the top chamber were both protected from infection (Fig. 8b, c). To determine whether the supernatants would protect activated T cells, we incubated CD3/CD28-activated CD4+ T cells with conditioned medium from drug-treated or untreated monocytes and infected them with HIV-1 GFP reporter virus. The results showed that supernatant from the R848-treated monocytes blocked infection of the activated CD4+ T cells (Fig. 8d). Supernatant produced by R848-treated activated CD4+ T cells did not protect monocytes from infection, demonstrating that the results with drug-treated monocytes were not caused by carry-over of residual R848 in the cultures (Fig. 8e). To determine whether the major inhibitory factor released by the monocytes was type-I IFN, we tested whether the addition of the IFN blocking antibody cocktail to the supernatant derived from R848-treated monocytes would neutralize its inhibitory effect. The results showed that the antibody cocktail neutralized its antiviral activity (Fig. 8f). We concluded that treatment of monocytes with R848 results in dual mechanism of HIV-1 restriction: the cell-intrinsic destabilization of the viral RNA, and a cell-extrinsic inhibition in which bystander monocytes and T cells are protected from infection as a result of type-I IFN produced by the treated monocytes.

## Discussion

We show that the TLR7/8 agonist R848 potently blocks HIV-1 replication by acting through two different mechanisms. It induces a post-entry block in monocytes that prevents reverse transcription and it causes monocytes to secrete type-I IFN that protects bystander T cells. The post-entry block results in a major reduction in the number of viral DNA reverse transcripts and destabilization of the viral genomic RNA. The restriction was independent of SAMHD1, a restriction factor in myeloid cells that also prevents reverse transcription. The R848-induced block to infection was active in mouse myeloid cells, which were also protected from infection. Because the mouse genome encodes a nonfunctional TLR8, this finding suggests that TLR7 activation is sufficient to induce the block to HIV-1 replication [19, 49, 50]. The effect was largely specific to monocytes as the drug had no effect on HIV-1 replication in activated T cells and a more minor effect on replication in MDM. R848 induced monocytes to secrete high levels of proinflammatory cytokines, including type-I IFN which protected bystander activated T cells from infection. Thus, the drug inhibits HIV-1 replication by two pathways, one in which a restriction is induced in the target monocytes and another in which cytokines are produced that protect bystander monocytes and T cells.

R848 treatment of monocytes was associated with a significant increase in the post-entry half-life of the viral genomic RNA, consistent with a direct inhibition of reverse transcription or a prior block to uncoating. The decreased stability of the viral genomic RNA could result from the induction of a host ribonuclease that attacks the reverse transcription complex, or, alternatively, from aberrant virion uncoating that subjects the viral genomic RNA molecules to attack by host ribonucleases. The effect was not the result of HIV-1 reverse transcriptase RNaseH, as the addition of nevirapine had no effect on viral genomic RNA half-life. In this analysis, we could not rule-out that a portion of the viral RNA measured was from virions that were either bound to the target cell plasma membrane or in endosomes; however, it remains the case that R848 treatment results in decreased stability of the viral RNA suggesting that much of the RNA measured originated from virus that had entered the cell.

Although SAMHD1 is known to inhibit HIV-1 reverse transcription in monocytes, our findings argue against a role for it in the R848-induced block to virus replication. The block to infection was strongly induced in mouse SAMHD1 knock-out BMDCs which are otherwise highly susceptible to HIV-1. In addition, the R848-induced block to infection of monocytes was not relieved by Vpx, which causes the rapid degradation of SAMHD1. R848 did not increase the expression level of SAMHD1 and did not interfere with the ability of Vpx to degrade SAMHD1. Furthermore, the block to infection is not consistent with any of the known monocyte-expressed restriction factors. MX2 acts post-reverse transcription, at nuclear import [51]; IFITMs act at entry [52]; SERINC3, SERINC5 [53, 54], REAF [55] and Lv2 [56] do not act on VSV-G pseudotyped virus. Moreover, virus produced in 293T cells is not significantly affected by SERINC3 and SERINC5 which are poorly expressed in 293T cells. Other known resrtriction factors are also unlikely to play a role in the restriction induced by R848. GBP5 and tetherin act post-integration [57, 58]. While Rhesus macaque TRIM5α destabilizes the HIV-1 capsid, human TRIM5α has little activity against the virus [59, 60]. In addition, while TRIM5α restriction is saturable, the R848 effect was not.

 Both, TLR7/8 and the cytoplasmic sensors MAVS and RIG-I play roles in sensing the RNA of several viruses [17, 18, 61–63]. While several reports suggest that TLR7/8 sense HIV-1 genomic RNA [19, 20], our findings argue against such a role in monocytes. TLR7/8 activation by R848 induces a strong block to reverse transcription in monocytes. If HIV-1 virions were to similarly activate these TLRs, the infection would be terminated, as is the case when the cells are treated with R848. This, however, was not the case: Vpx-containing viruses infected the monocytes to high levels. R848 prevented HIV-1 infection when added prior to, or at the same time as the virus, demonstrating that the block to infection is activated rapidly upon TLR7/8 activation and is capable of terminating the infection. The ability of HIV-1 to avoid activating the TLRs suggest that the virus has evolved a strategy to shield its RNA from sensing, as is the case for HIV-1 DNA reverse transcripts which are shielded from the cytoplasmic double stranded DNA sensor cGAS by the viral capsid [64, 65].

A previous study by Wang et al. [8] showed that LPS, R848 and double-stranded RNA induced a post-entry block to HIV reverse transcription in MDMs but not activated T cells. Here, we measured single-cycle reporter gene expression rather than released p24 and used undifferentiated monocytes rather than MDMs. In addition, they found that LPS treatment of MDMs induced the secretion of an antiviral factor that was not IFNβ. In our experiments, the primary soluble inhibitory factor induced by R848-treated monocytes was type-I IFN. These differences may be due to the use of Vpx-containing virus that efficiently infects myeloid cells to induce high levels of cytokine production. Alternatively, monocytes may respond more strongly to TLR activation than MDMs.

Buitendijk et al. [7] demonstrated that TLR7 and TLR8 activation inhibit HIV-1 replication. Treatment of MDMs with the TLR7 agonist gardiquimod induced a block to infection of co-cultured, activated PBMC and treatment of activated PBMC with agonists specific for TLRs 3, 7, 8 and 9 blocked HIV-1 replication [10]. In both cases, the block was to infection of stimulated PBMCs where the major target cells targeted for HIV-1 infection are activated T cells. We and Wang et al. [8] find that TLR7/8 agonist treatment of activated T cells has no effect on HIV-1. Thus, the R848-induced restriction in stimulated PBMC is mediated by a soluble factor such as type-I IFN released by MDMs, monocytes or pDC, while the post-entry block in a single-cycle infection is due to an intrinsic restriction and not a soluble factor.

TLR agonists are under development for clinical use in attacking the latent HIV-1 reservoir [66]. Such agonists stimulate transcription of latent proviruses in resting T cells without activating the cell [67–71]. Such agonists would have the added advantages that they induce a potent block to the replication of HIV-1 in myeloid cells and induce the production of type-I IFN that protects CD4 T cells from infection. In addition, the induced production of cytokines such as TNFα by monocytes would activate proviruses in latently infected cells. While the  treatment of patients with TLR agonists may be accompanied by inflammatory responses including lymphopenia, elevated cytokines and splenomegaly as is the case in mice treated with R848, agonists under development may be less inflammatory.

## Conclusion

We show here that R848 induces two independent block to HIV-1 replication. The first, is a cell-intrinsic block that acts in monocytes to prevent reverse transcription, most likely by inducing a novel restriction factor. The restriction was associated with the post-entry destabilization of the HIV-1 genomic RNA, possibly caused by the induction of a ribonuclease or by a factor that interferes with capsid uncoating. The second, is a cell-extrinsic block caused by the R848-induced release of proinflammatory cytokines, primarily type-I IFN, by monocytes that protects both bystander monocytes and CD4+ T cells. The fact that the restriction was not induced by HIV-1 infection itself argues that the viral RNA is shielded upon uncoating, preventing sensing in monocytes by TLR7/8. These findings highlight the existence of yet unidentified host restriction factors and suggest that the activity identified here may constitute “Lv5”.

## Additional files

**Additional file 1.** R848 blocks Vpx-containing HIV-1 infection from multiple donors. PBMC from healthy donors were pretreated with 10 μM R848 (*blue squares*) for 24 h and then infected with HIV1 X+ luciferase reporter virus. Infectivity was measured 72 h post infection. The data are from independent experiments with 18 different donors (***p* = 0.0011).

**Additional file 2.** R848 is not cytotoxic. **a** Cell counts of trypan blue stained human primary monocytes (2 Donors) 48 h post-treatment with the indicated concentration of R848. **b** Cell viability of monocytes isolated from 2 Donors as determined by cell titer assay (Promega) 48 h post-treatment with indicated concentration of R848 (*blue*). Triton X-100 (TX-100) is a control for dead cells. **c**, **d** Viability of R848- or IFNα-treated primary human monocytes (**c**) or R848-treated BMDDC from SAMHD1 knock-out mice (**d**) was determined 96 h post treatment by flow cytometry using efluor PacBlue viability stain (eBiosciences).

**Additional file 3.** The R848-induced block is potent. Two additional donors, (**a**) and (**b**), similar to Fig. 2a are shown. Monocytes were incubated for 24 h with 1 µM (*dark blue*) or 10 µM (*light blue*) R848 and then infected with increasing amounts of HIV1 X+ luciferase reporter virus (0.1, 0.3, 1.0 and 3.0 × 10^6^ cps). Uninfected (mock) and cells infected with HIV-1 luciferase virus lacking Vpx (HIV1) are included as controls.

**Additional file 4.** R848 does not induce APOBEC3A expression in monocytes. Monocytes from 3 healthy donors were treated with 10 μM R848, 100 U/mL IFNαor untreated (mock). After 24 h, the cells were lysed and the lysates analyzed on an immunoblot probed with anti-APOBEC3A (A3A) and anti-tubulin antibody.

**Additional file 5.** R848 causes the release of pro-inflammatory cytokines. Two additional donors, **a** and **b**, similar to Fig. 4c are shown. Monocytes were incubated with indicated concentrations of R848 and the supernatant was collected 24 h post-treatment. Cytokines in the supernatants were quantified by cytokine bead array (BD biosciences).

**Additional file 6.** Recombinant IL-1β does not block HIV infection in PBMC. Two additional donors similar to Fig. 4e are shown. PBMC from 2 healthy donors were treated with 100 U/mL IFNα, 10 μM R848 or 0.1, 1, 10 or 100 ng/mL IL-1μ for 24 h. The cells were then infected with HIV1 or HIV1 X+ luciferase reporter virus and infectivity was measured 72 h post-infection by luciferase assay.

## References

[1] Akira S, Takeda K (2004). Toll-like receptor signalling. Nat Rev Immunol.

[2] Takeda K, Akira S (2005). Toll-like receptors in innate immunity. Int Immunol.

[3] Xagorari A, Chlichlia K (2008). Toll-like receptors and viruses: induction of innate antiviral immune responses. Open Microbiol J.

[4] Boivin N, Sergerie Y, Rivest S, Boivin G (2008). Effect of pretreatment with toll-like receptor agonists in a mouse model of herpes simplex virus type 1 encephalitis. J Infect Dis.

[5] Sariol CA, Martinez MI, Rivera F, Rodriguez IV, Pantoja P, Abel K, Arana T, Giavedoni L, Hodara V, White LJ (2011). Decreased dengue replication and an increased anti-viral humoral response with the use of combined Toll-like receptor 3 and 7/8 agonists in macaques. PLoS ONE.

[6] Zhang X, Chentoufi AA, Dasgupta G, Nesburn AB, Wu M, Zhu X, Carpenter D, Wechsler SL, You S, BenMohamed L (2009). A genital tract peptide epitope vaccine targeting TLR-2 efficiently induces local and systemic CD8+ T cells and protects against herpes simplex virus type 2 challenge. Mucosal Immunol.

[7] Buitendijk M, Eszterhas SK, Howell AL (2013). Gardiquimod: a Toll-like receptor-7 agonist that inhibits HIV type 1 infection of human macrophages and activated T cells. AIDS Res Hum Retrovir.

[8] Wang X, Chao W, Saini M, Potash MJ (2011). A common path to innate immunity to HIV-1 induced by Toll-like receptor ligands in primary human macrophages. PLoS ONE.

[9] Nian H, Geng WQ, Cui HL, Bao MJ, Zhang ZN, Zhang M, Pan Y, Hu QH, Shang H (2012). R-848 triggers the expression of TLR7/8 and suppresses HIV replication in monocytes. BMC Infect Dis.

[10] Buitendijk M, Eszterhas SK, Howell AL (2014). Toll-like receptor agonists are potent inhibitors of human immunodeficiency virus-type 1 replication in peripheral blood mononuclear cells. AIDS Res Hum Retrovir.

[11] Gotovtseva EP, Kapadia AS, Smolensky MH, Lairson DR (2008). Optimal frequency of imiquimod (aldara) 5% cream for the treatment of external genital warts in immunocompetent adults: a meta-analysis. Sex Transm Dis.

[12] Mark KE, Corey L, Meng TC, Magaret AS, Huang ML, Selke S, Slade HB, Tyring SK, Warren T, Sacks SL (2007). Topical resiquimod 0.01% gel decreases herpes simplex virus type 2 genital shedding: a randomized, controlled trial. J Infect Dis.

[13] Altfeld M, Gale M (2015). Innate immunity against HIV-1 infection. Nat Immunol.

[14] Mandl JN, Barry AP, Vanderford TH, Kozyr N, Chavan R, Klucking S, Barrat FJ, Coffman RL, Staprans SI, Feinberg MB (2008). Divergent TLR7 and TLR9 signaling and type I interferon production distinguish pathogenic and nonpathogenic AIDS virus infections. Nat Med.

[15] O’Brien M, Manches O, Sabado RL, Baranda SJ, Wang Y, Marie I, Rolnitzky L, Markowitz M, Margolis DM, Levy D (2011). Spatiotemporal trafficking of HIV in human plasmacytoid dendritic cells defines a persistently IFN-alpha-producing and partially matured phenotype. J Clin Investig.

[16] O’Brien M, Manches O, Wilen C, Gopal R, Huq R, Wu V, Sunseri N, Bhardwaj N (2016). CD4 receptor is a key determinant of divergent HIV-1 sensing by plasmacytoid dendritic cells. PLoS Pathog.

[17] Chattergoon MA, Latanich R, Quinn J, Winter ME, Buckheit RW, Blankson JN, Pardoll D, Cox AL (2014). HIV and HCV activate the inflammasome in monocytes and macrophages via endosomal Toll-like receptors without induction of type 1 interferon. PLoS Pathog.

[18] Guo H, Gao J, Taxman DJ, Ting JP, Su L (2014). HIV-1 infection induces interleukin-1beta production via TLR8 protein-dependent and NLRP3 inflammasome mechanisms in human monocytes. J Biol Chem.

[19] Heil F, Hemmi H, Hochrein H, Ampenberger F, Kirschning C, Akira S, Lipford G, Wagner H, Bauer S (2004). Species-specific recognition of single-stranded RNA via toll-like receptor 7 and 8. Science.

[20] Meier A, Bagchi A, Sidhu HK, Alter G, Suscovich TJ, Kavanagh DG, Streeck H, Brockman MA, LeGall S, Hellman J (2008). Upregulation of PD-L1 on monocytes and dendritic cells by HIV-1 derived TLR ligands. Aids.

[21] Simmons RP, Scully EP, Groden EE, Arnold KB, Chang JJ, Lane K, Lifson J, Rosenberg E, Lauffenburger DA, Altfeld M (2013). HIV-1 infection induces strong production of IP-10 through TLR7/9-dependent pathways. Aids.

[22] Nazli A, Kafka JK, Ferreira VH, Anipindi V, Mueller K, Osborne BJ, Dizzell S, Chauvin S, Mian MF, Ouellet M (2013). HIV-1 gp120 induces TLR2- and TLR4-mediated innate immune activation in human female genital epithelium. J Immunol.

[23] Reuven EM, Ali M, Rotem E, Schwarzer R, Gramatica A, Futerman AH, Shai Y (2014). The HIV-1 envelope transmembrane domain binds TLR2 through a distinct dimerization motif and inhibits TLR2-mediated responses. PLoS Pathog.

[24] Gartner S, Markovits P, Markovitz DM, Kaplan MH, Gallo RC, Popovic M (1986). The role of mononuclear phagocytes in HTLV-III/LAV infection. Science.

[25] Hrecka K, Hao C, Gierszewska M, Swanson SK, Kesik-Brodacka M, Srivastava S, Florens L, Washburn MP, Skowronski J (2011). Vpx relieves inhibition of HIV-1 infection of macrophages mediated by the SAMHD1 protein. Nature.

[26] Laguette N, Sobhian B, Casartelli N, Ringeard M, Chable-Bessia C, Segeral E, Yatim A, Emiliani S, Schwartz O, Benkirane M (2011). SAMHD1 is the dendritic- and myeloid-cell-specific HIV-1 restriction factor counteracted by Vpx. Nature.

[27] Lahouassa H, Daddacha W, Hofmann H, Ayinde D, Logue EC, Dragin L, Bloch N, Maudet C, Bertrand M, Gramberg T (2012). SAMHD1 restricts the replication of human immunodeficiency virus type 1 by depleting the intracellular pool of deoxynucleoside triphosphates. Nat Immunol.

[28] Brandariz-Nunez A, Valle-Casuso JC, White TE, Laguette N, Benkirane M, Brojatsch J, Diaz-Griffero F (2012). Role of SAMHD1 nuclear localization in restriction of HIV-1 and SIVmac. Retrovirology.

[29] Fujita M, Otsuka M, Miyoshi M, Khamsri B, Nomaguchi M, Adachi A (2008). Vpx is critical for reverse transcription of the human immunodeficiency virus type 2 genome in macrophages. J Virol.

[30] Goujon C, Jarrosson-Wuilleme L, Bernaud J, Rigal D, Darlix JL, Cimarelli A (2006). With a little help from a friend: increasing HIV transduction of monocyte-derived dendritic cells with virion-like particles of SIV(MAC). Gene Ther.

[31] Goujon C, Riviere L, Jarrosson-Wuilleme L, Bernaud J, Rigal D, Darlix JL, Cimarelli A (2007). SIVSM/HIV-2 Vpx proteins promote retroviral escape from a proteasome-dependent restriction pathway present in human dendritic cells. Retrovirology.

[32] Hofmann H, Logue EC, Bloch N, Daddacha W, Polsky SB, Schultz ML, Kim B, Landau NR (2012). The Vpx lentiviral accessory protein targets SAMHD1 for degradation in the nucleus. J Virol.

[33] Schule S, Kloke BP, Kaiser JK, Heidmeier S, Panitz S, Wolfrum N, Cichutek K, Schweizer M (2009). Restriction of HIV-1 replication in monocytes is abolished by Vpx of SIVsmmPBj. PLoS ONE.

[34] Wolfrum N, Muhlebach MD, Schule S, Kaiser JK, Kloke BP, Cichutek K, Schweizer M (2007). Impact of viral accessory proteins of SIVsmmPBj on early steps of infection of quiescent cells. Virology.

[35] Accola MA, Bukovsky AA, Jones MS, Gottlinger HG (1999). A conserved dileucine-containing motif in p6(gag) governs the particle association of Vpx and Vpr of simian immunodeficiency viruses SIV(mac) and SIV(agm). J Virol.

[36] Sunseri N, O’Brien M, Bhardwaj N, Landau NR (2011). Human immunodeficiency virus type 1 modified to package Simian immunodeficiency virus Vpx efficiently infects macrophages and dendritic cells. J Virol.

[37] Dull T, Zufferey R, Kelly M, Mandel RJ, Nguyen M, Trono D, Naldini L (1998). A third-generation lentivirus vector with a conditional packaging system. J Virol.

[38] Keele BF, Giorgi EE, Salazar-Gonzalez JF, Decker JM, Pham KT, Salazar MG, Sun C, Grayson T, Wang S, Li H (2008). Identification and characterization of transmitted and early founder virus envelopes in primary HIV-1 infection. Proc Natl Acad Sci USA.

[39] Hofmann H, Norton TD, Schultz ML, Polsky SB, Sunseri N, Landau NR (2013). Inhibition of CUL4A Neddylation causes a reversible block to SAMHD1-mediated restriction of HIV-1. J Virol.

[40] Butler SL, Hansen MS, Bushman FD (2001). A quantitative assay for HIV DNA integration in vivo. Nat Med.

[41] Uze G, Di Marco S, Mouchel-Vielh E, Monneron D, Bandu MT, Horisberger MA, Dorques A, Lutfalla G, Mogensen KE (1994). Domains of interaction between alpha interferon and its receptor components. J Mol Biol.

[42] Baldauf HM, Pan X, Erikson E, Schmidt S, Daddacha W, Burggraf M, Schenkova K, Ambiel I, Wabnitz G, Gramberg T (2012). SAMHD1 restricts HIV-1 infection in resting CD4(+) T cells. Nat Med.

[43] Descours B, Cribier A, Chable-Bessia C, Ayinde D, Rice G, Crow Y, Yatim A, Schwartz O, Laguette N, Benkirane M (2012). SAMHD1 restricts HIV-1 reverse transcription in quiescent CD4(+) T-cells. Retrovirology.

[44] Cavrois M, De Noronha C, Greene WC (2002). A sensitive and specific enzyme-based assay detecting HIV-1 virion fusion in primary T lymphocytes. Nat Biotechnol.

[45] Berger G, Durand S, Fargier G, Nguyen XN, Cordeil S, Bouaziz S, Muriaux D, Darlix JL, Cimarelli A (2011). APOBEC3A is a specific inhibitor of the early phases of HIV-1 infection in myeloid cells. PLoS Pathog.

[46] Berger A, Munk C, Schweizer M, Cichutek K, Schule S, Flory E (2010). Interaction of Vpx and apolipoprotein B mRNA-editing catalytic polypeptide 3 family member A (APOBEC3A) correlates with efficient lentivirus infection of monocytes. J Biol Chem.

[47] Allouch A, David A, Amie SM, Lahouassa H, Chartier L, Margottin-Goguet F, Barre-Sinoussi F, Kim B, Saez-Cirion A, Pancino G (2013). p21-mediated RNR2 repression restricts HIV-1 replication in macrophages by inhibiting dNTP biosynthesis pathway. Proc Natl Acad Sci USA.

[48] Pauls E, Ruiz A, Riveira-Munoz E, Permanyer M, Badia R, Clotet B, Keppler OT, Ballana E, Este JA (2014). p21 regulates the HIV-1 restriction factor SAMHD1. Proc Natl Acad Sci USA.

[49] Hemmi H, Kaisho T, Takeuchi O, Sato S, Sanjo H, Hoshino K, Horiuchi T, Tomizawa H, Takeda K, Akira S (2002). Small anti-viral compounds activate immune cells via the TLR7 MyD88-dependent signaling pathway. Nat Immunol.

[50] Liu J, Xu C, Hsu LC, Luo Y, Xiang R, Chuang TH (2010). A five-amino-acid motif in the undefined region of the TLR8 ectodomain is required for species-specific ligand recognition. Mol Immunol.

[51] Goujon C, Moncorge O, Bauby H, Doyle T, Ward CC, Schaller T, Hue S, Barclay WS, Schulz R, Malim MH (2013). Human MX2 is an interferon-induced post-entry inhibitor of HIV-1 infection. Nature.

[52] Lu J, Pan Q, Rong L, He W, Liu SL, Liang C (2011). The IFITM proteins inhibit HIV-1 infection. J Virol.

[53] Rosa A, Chande A, Ziglio S, De Sanctis V, Bertorelli R, Goh SL, McCauley SM, Nowosielska A, Antonarakis SE, Luban J (2015). HIV-1 Nef promotes infection by excluding SERINC5 from virion incorporation. Nature.

[54] Usami Y, Wu Y, Gottlinger HG (2015). SERINC3 and SERINC5 restrict HIV-1 infectivity and are counteracted by Nef. Nature.

[55] Marno KM, Ogunkolade BW, Pade C, Oliveira NM, O’Sullivan E, McKnight A (2014). Novel restriction factor RNA-associated early-stage anti-viral factor (REAF) inhibits human and simian immunodeficiency viruses. Retrovirology.

[56] McKnight A, Griffiths DJ, Dittmar M, Clapham P, Thomas E (2001). Characterization of a late entry event in the replication cycle of human immunodeficiency virus type 2. J Virol.

[57] Krapp C, Hotter D, Gawanbacht A, McLaren PJ, Kluge SF, Sturzel CM, Mack K, Reith E, Engelhart S, Ciuffi A (2016). Guanylate Binding Protein (GBP) 5 Is an Interferon-Inducible Inhibitor of HIV-1 Infectivity. Cell Host Microbe.

[58] Neil SJ, Zang T, Bieniasz PD (2008). Tetherin inhibits retrovirus release and is antagonized by HIV-1 Vpu. Nature.

[59] Stremlau M, Owens CM, Perron MJ, Kiessling M, Autissier P, Sodroski J (2004). The cytoplasmic body component TRIM5alpha restricts HIV-1 infection in Old World monkeys. Nature.

[60] Stremlau M, Perron M, Welikala S, Sodroski J (2005). Species-specific variation in the B30.2(SPRY) domain of TRIM5alpha determines the potency of human immunodeficiency virus restriction. J Virol.

[61] Berg RK, Melchjorsen J, Rintahaka J, Diget E, Soby S, Horan KA, Gorelick RJ, Matikainen S, Larsen CS, Ostergaard L (2012). Genomic HIV RNA induces innate immune responses through RIG-I-dependent sensing of secondary-structured RNA. PLoS ONE.

[62] Seth RB, Sun L, Ea CK, Chen ZJ (2005). Identification and characterization of MAVS, a mitochondrial antiviral signaling protein that activates NF-kappaB and IRF 3. Cell.

[63] Solis M, Nakhaei P, Jalalirad M, Lacoste J, Douville R, Arguello M, Zhao T, Laughrea M, Wainberg MA, Hiscott J (2011). RIG-I-mediated antiviral signaling is inhibited in HIV-1 infection by a protease-mediated sequestration of RIG-I. J Virol.

[64] Lahaye X, Satoh T, Gentili M, Cerboni S, Conrad C, Hurbain I, El Marjou A, Lacabaratz C, Lelievre JD, Manel N (2013). The capsids of HIV-1 and HIV-2 determine immune detection of the viral cDNA by the innate sensor cGAS in dendritic cells. Immunity.

[65] Rasaiyaah J, Tan CP, Fletcher AJ, Price AJ, Blondeau C, Hilditch L, Jacques DA, Selwood DL, James LC, Noursadeghi M (2013). HIV-1 evades innate immune recognition through specific cofactor recruitment. Nature.

[66] Offersen R, Nissen SK, Rasmussen TA, Ostergaard L, Denton PW, Sogaard OS, Tolstrup M (2016). A novel Toll-like receptor 9 agonist, MGN1703, enhances HIV-1 transcription and NK cell-mediated inhibition of HIV-1-infected autologous CD4+ T cells. J Virol.

[67] Novis CL, Archin NM, Buzon MJ, Verdin E, Round JL, Lichterfeld M, Margolis DM, Planelles V, Bosque A (2013). Reactivation of latent HIV-1 in central memory CD4(+) T cells through TLR-1/2 stimulation. Retrovirology.

[68] Scheller C, Ullrich A, McPherson K, Hefele B, Knoferle J, Lamla S, Olbrich AR, Stocker H, Arasteh K, ter Meulen V (2004). CpG oligodeoxynucleotides activate HIV replication in latently infected human T cells. J Biol Chem.

[69] Schlaepfer E, Audige A, Joller H, Speck RF (2006). TLR7/8 triggering exerts opposing effects in acute versus latent HIV infection. J Immunol.

[70] Schlaepfer E, Speck RF (2011). TLR8 activates HIV from latently infected cells of myeloid-monocytic origin directly via the MAPK pathway and from latently infected CD4+ T cells indirectly via TNF-alpha. J Immunol.

[71] Thibault S, Imbeault M, Tardif MR, Tremblay MJ (2009). TLR5 stimulation is sufficient to trigger reactivation of latent HIV-1 provirus in T lymphoid cells and activate virus gene expression in central memory CD4+ T cells. Virology.

