# Supplementary figures and images for: TLR7/8 agonist induces a post-entry SAMHD1-independent block to HIV-1 infection of monocytes
Source: Retrovirology. 2016 Dec 1;13:83. doi: 10.1186/s12977-016-0316-3 (PMC5131500; doi:10.1186/s12977-016-0316-3)

## Slide 1
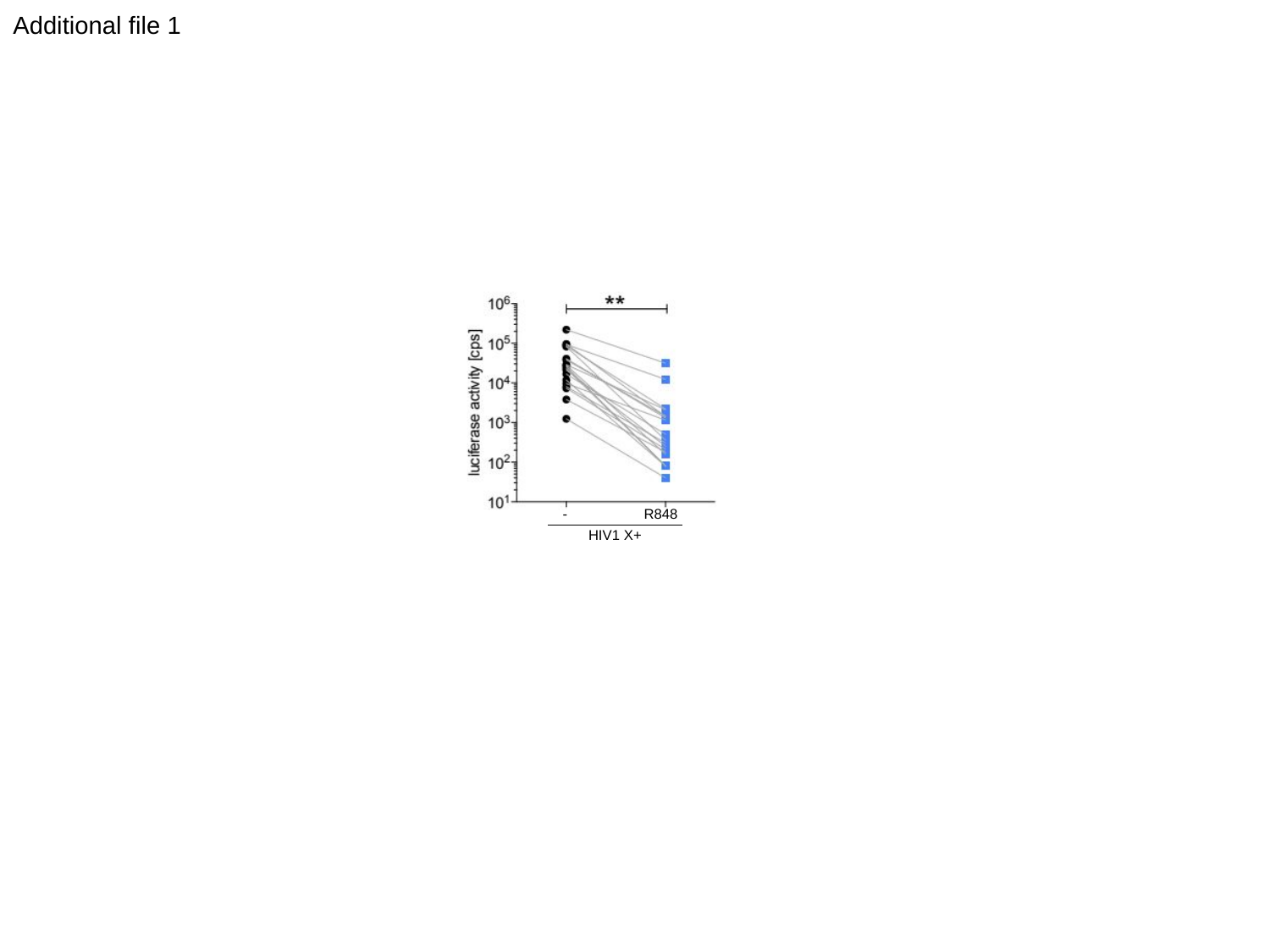

Additional file 1
R848
-
HIV1 X+

Supplement: Supplementary file 1 — Additional file 1. R848 blocks Vpx-containing HIV-1 infection from multiple donors. PBMC from healthy donors were pretreated with 10 μM R848 (blue squares) for 24 h and then infected with HIV1 X+ luciferase reporter virus. Infectivity was measured 72 h post infection. The data are from independent experiments with 18 different donors (**p = 0.0011). [file 12977_2016_316_MOESM1_ESM.pptx]

## Slide 1
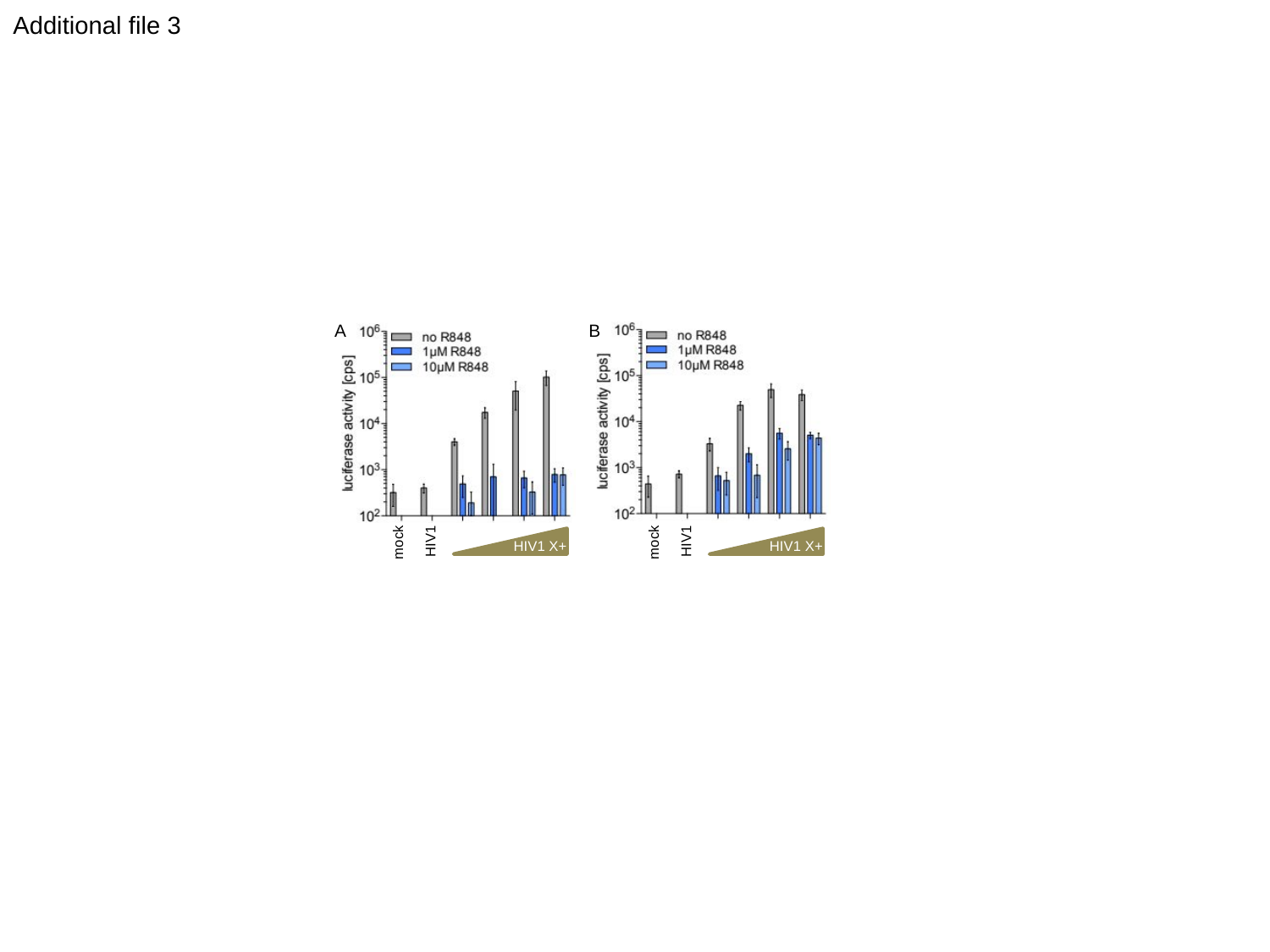

Additional file 3
A
B
HIV1
HIV1
mock
mock
HIV1 X+
HIV1 X+

Supplement: Supplementary file 3 — Additional file 3. The R848-induced block is potent. Two additional donors, (a) and (b), similar to Fig. 2a are shown. Monocytes were incubated for 24 h with 1 µM (dark blue) or 10 µM (light blue) R848 and then infected with increasing amounts of HIV1 X+ luciferase reporter virus (0.1, 0.3, 1.0 and 3.0 × 10^6^ cps). Uninfected (mock) and cells infected with HIV-1 luciferase virus lacking Vpx (HIV1) are included as controls. [file 12977_2016_316_MOESM3_ESM.pptx]

## Slide 1
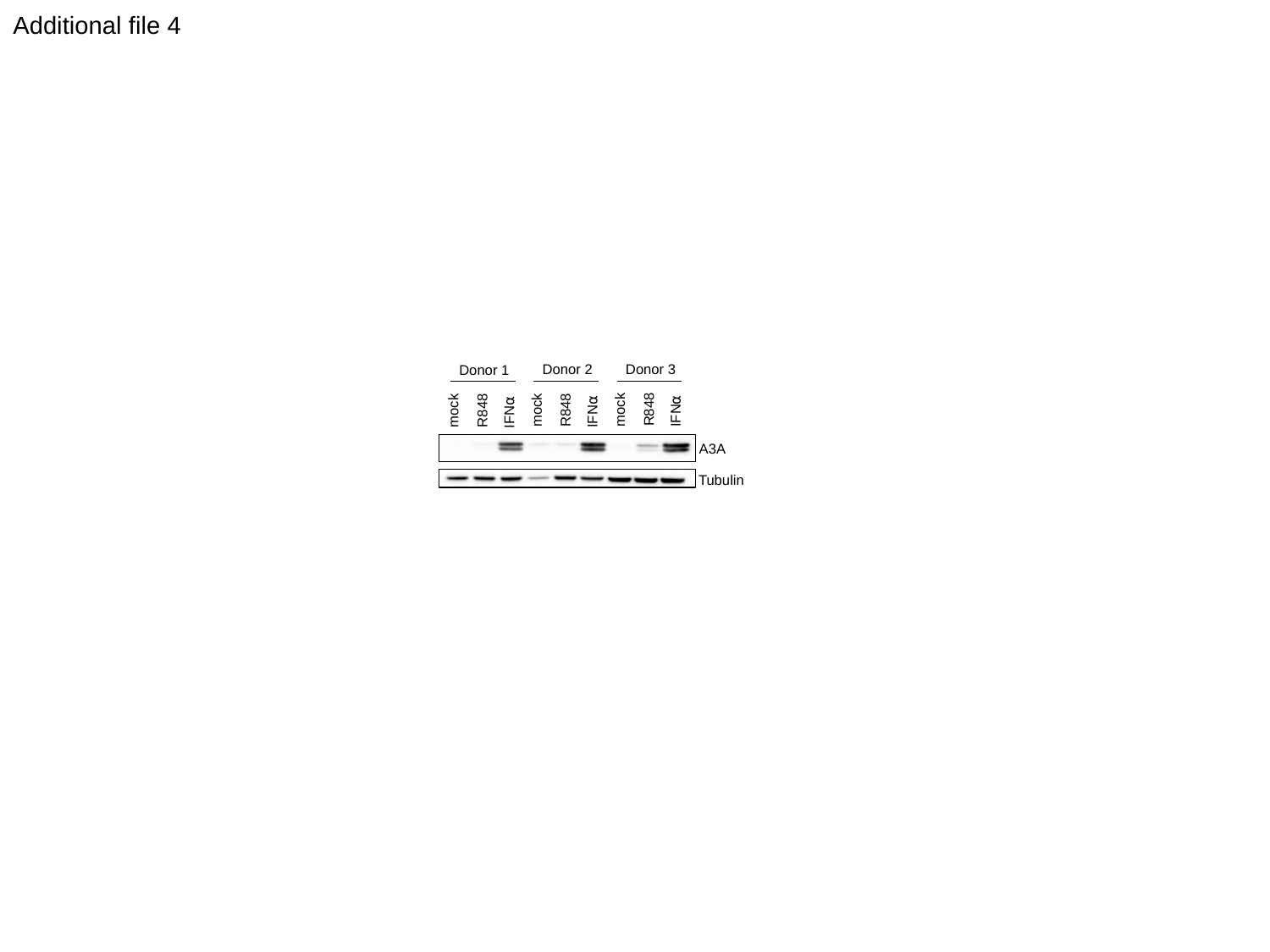

Additional file 4
Donor 3
Donor 2
Donor 1
mock
R848
mock
R848
mock
R848
IFN⍺
IFN⍺
IFN⍺
A3A
Tubulin

Supplement: Supplementary file 4 — Additional file 4. R848 does not induce APOBEC3A expression in monocytes. Monocytes from 3 healthy donors were treated with 10 μM R848, 100 U/mL IFNαor untreated (mock). After 24 h, the cells were lysed and the lysates analyzed on an immunoblot probed with anti-APOBEC3A (A3A) and anti-tubulin antibody. [file 12977_2016_316_MOESM4_ESM.pptx]

## Slide 1
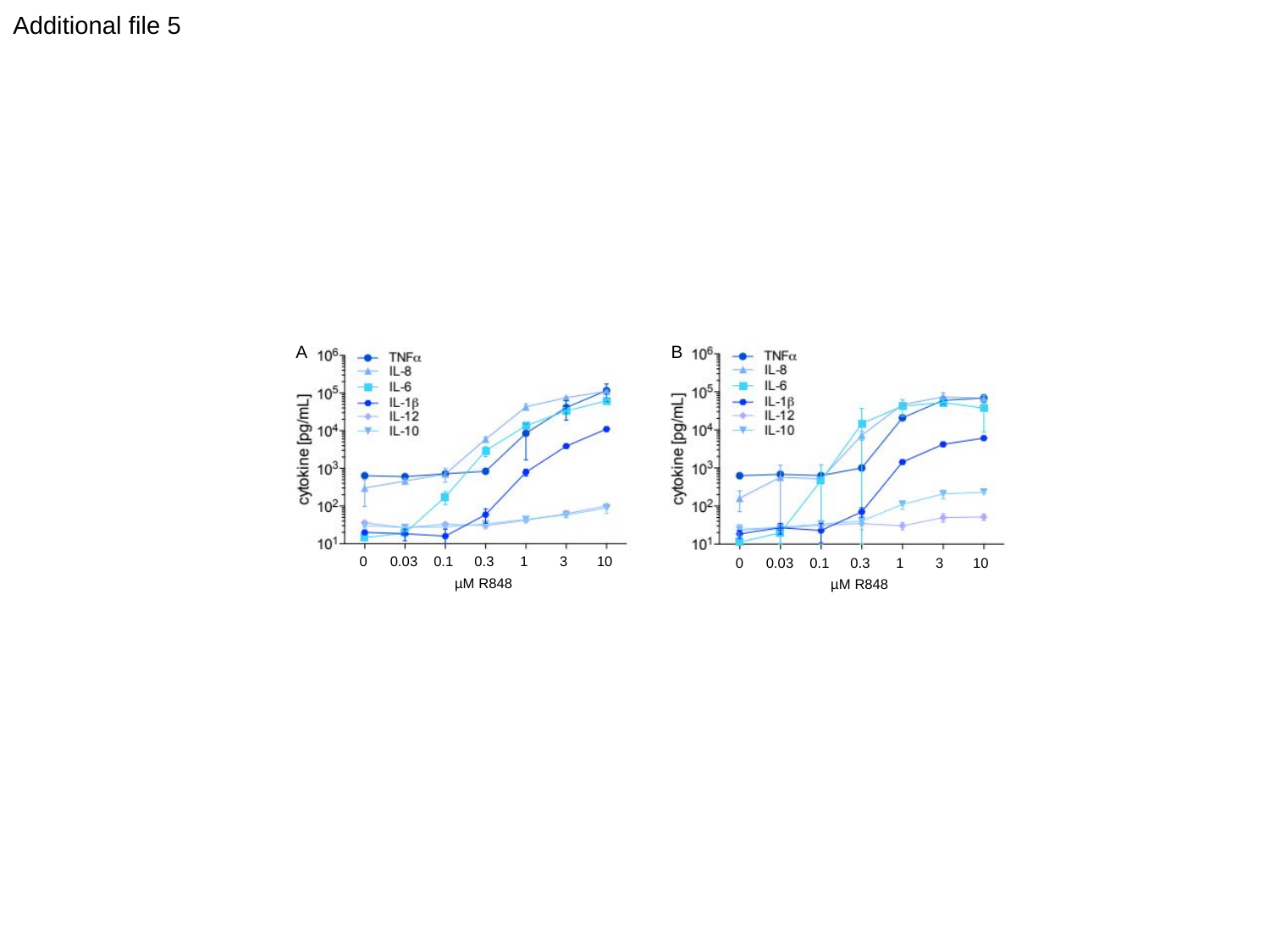

Additional file 5
A
B
0
0.03
0.1
0.3
1
3
10
0
0.03
0.1
0.3
1
3
10
µM R848
µM R848

Supplement: Supplementary file 5 — Additional file 5. R848 causes the release of pro-inflammatory cytokines. Two additional donors, a and b, similar to Fig. 4c are shown. Monocytes were incubated with indicated concentrations of R848 and the supernatant was collected 24 h post-treatment. Cytokines in the supernatants were quantified by cytokine bead array (BD biosciences). [file 12977_2016_316_MOESM5_ESM.pptx]

## Slide 1
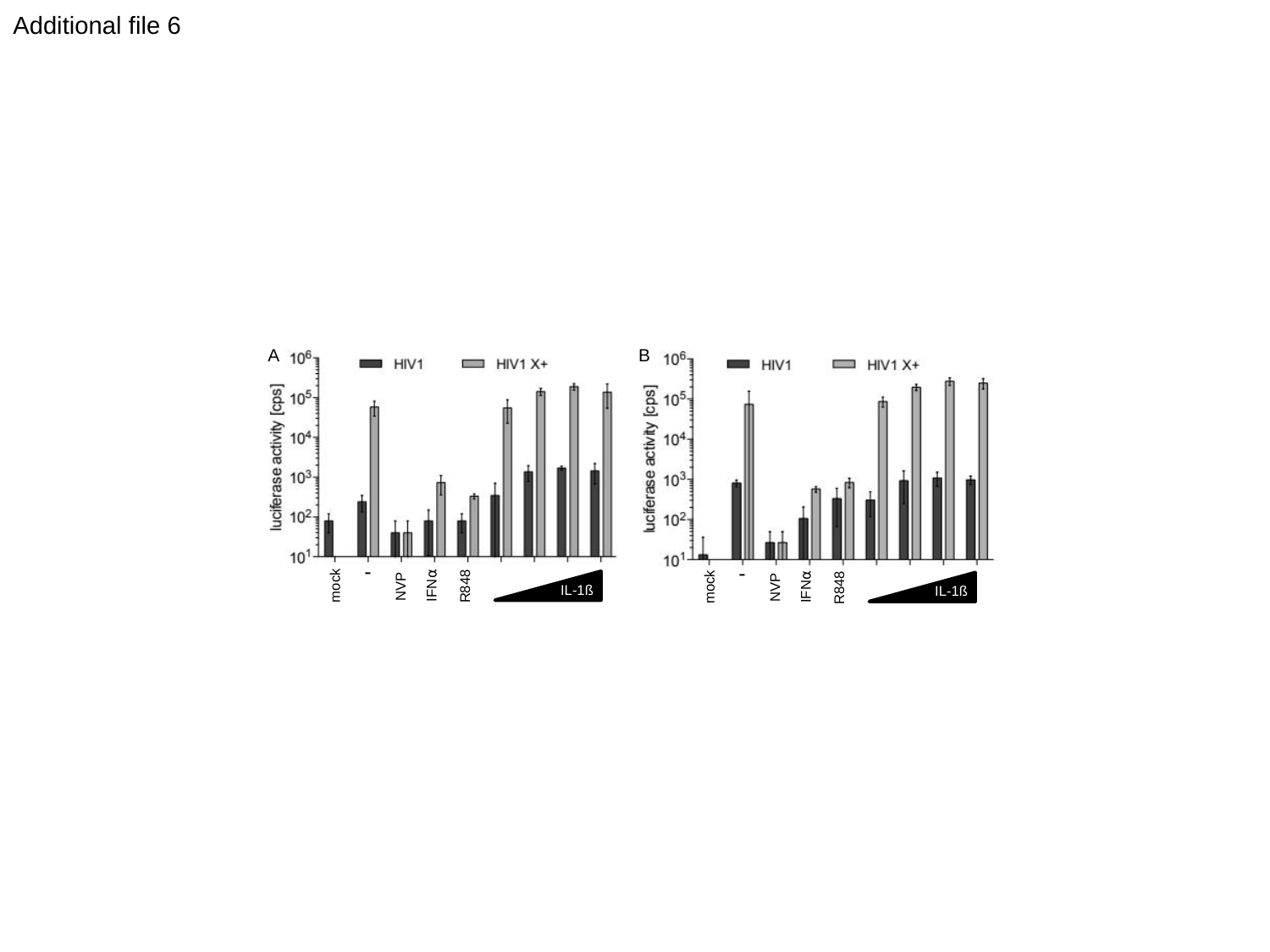

Additional file 6
A
B
-
-
mock
IFN⍺
NVP
R848
mock
IFN⍺
NVP
R848
IL-1ß
IL-1ß

Supplement: Supplementary file 6 — Additional file 6. Recombinant IL-1β does not block HIV infection in PBMC. Two additional donors similar to Fig. 4e are shown. PBMC from 2 healthy donors were treated with 100 U/mL IFNα, 10 μM R848 or 0.1, 1, 10 or 100 ng/mL IL-1μ for 24 h. The cells were then infected with HIV1 or HIV1 X+ luciferase reporter virus and infectivity was measured 72 h post-infection by luciferase assay. [file 12977_2016_316_MOESM6_ESM.pptx]
