# Supplementary material for: TLR7/8 agonist induces a post-entry SAMHD1-independent block to HIV-1 infection of monocytes
Source: Retrovirology. 2016 Dec 1;13:83. doi: 10.1186/s12977-016-0316-3 (PMC5131500; doi:10.1186/s12977-016-0316-3)
Supplement: Supplementary file 2 — Additional file 2. R848 is not cytotoxic. a Cell counts of trypan blue stained human primary monocytes (2 Donors) 48 h post-treatment with the indicated concentration of R848. b Cell viability of monocytes isolated from 2 Donors as determined by cell titer assay (Promega) 48 h post-treatment with indicated concentration of R848 (blue). Triton X-100 (TX-100) is a control for dead cells. c, d Viability of R848- or IFNα-treated primary human monocytes (c) or R848-treated BMDDC from SAMHD1 knock-out mice (d) was determined 96 h post treatment by flow cytometry using efluor PacBlue viability stain (eBiosciences). [file 12977_2016_316_MOESM2_ESM.pptx]

## Slide 1
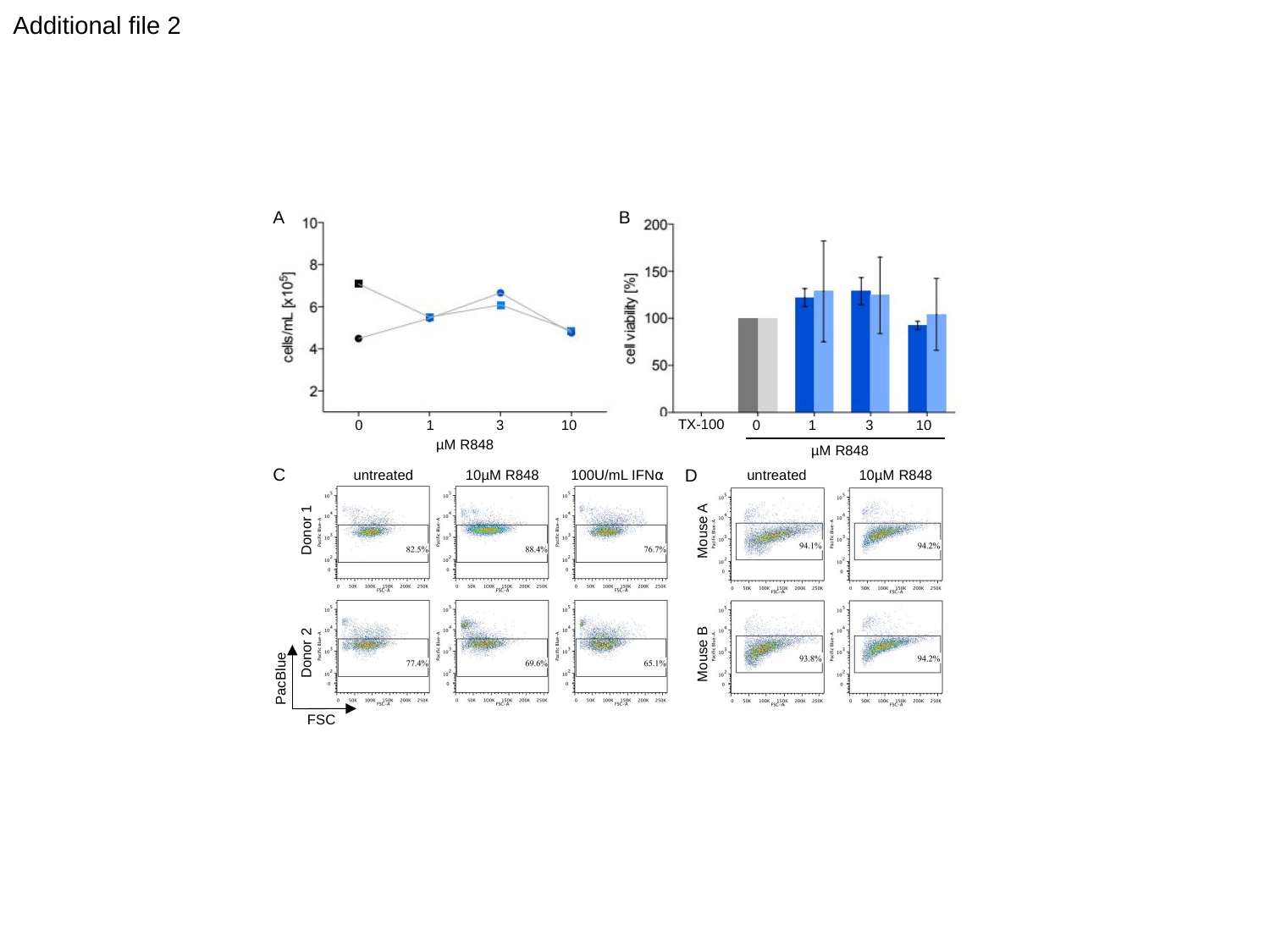

Additional file 2
A
B
TX-100
0
1
3
10
0
1
3
10
µM R848
µM R848
C
D
untreated
10µM R848
100U/mL IFN⍺
untreated
10µM R848
Donor 1
Mouse A
Donor 2
Mouse B
PacBlue
FSC
